# Supplementary material for: Exploring the roles of roughness, friction and adhesion in discontinuous shear thickening by means of thermo-responsive particles
Source: Nat Commun. 2021 Mar 5;12:1477. doi: 10.1038/s41467-021-21580-y (PMC7935878; doi:10.1038/s41467-021-21580-y)
Supplement: Supplementary file 1 — Supplementary Information [file 41467_2021_21580_MOESM1_ESM.pdf]

# Supplementary Information for Exploring the roles of roughness, friction and adhesion in discontinuous shear thickening by means of thermo-responsive particles

Chiao-Peng Hsu,[1,2] Joydeb Mandal,[2] Shivaprakash N. Ramakrishna,[2] Nicholas D. Spencer,[2] and Lucio Isa[1]

[1]*Laboratory for Soft Materials and Interfaces, Department of Materials, ETH Zurich, Zurich, Switzerland.*

[2]*Laboratory for Surface Science and Technology, Department of Materials, ETH Zurich, Zurich, Switzerland.*

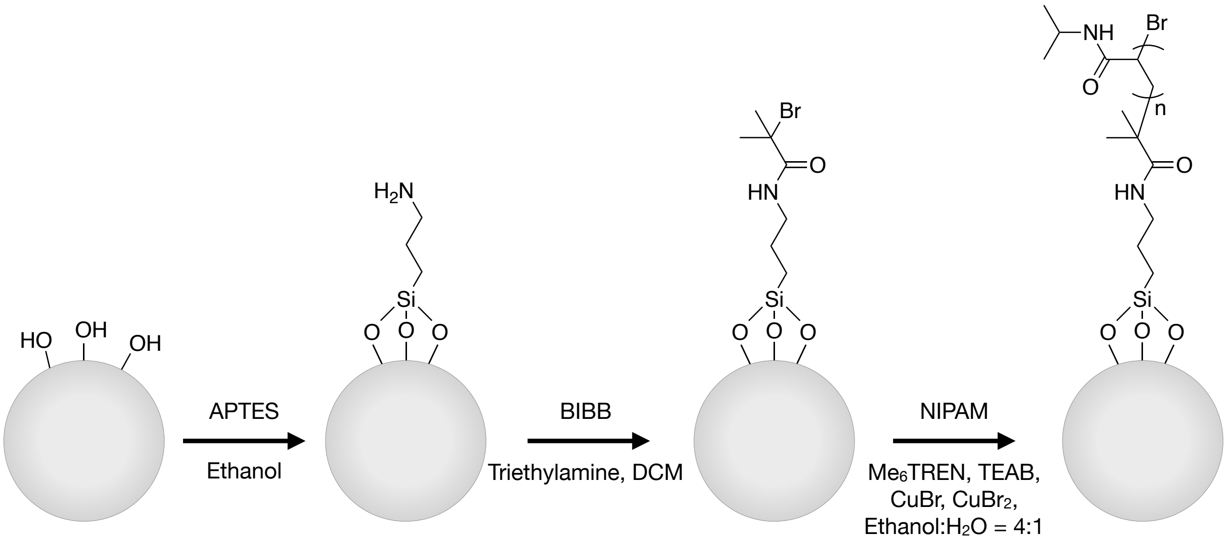


Supplementary Figure 1. **Reaction scheme to prepare PNIPAM brushes on the surface of a silica particle using SI-ATRP.** The silica particles are fictionalized with APTES and then grafted with the initiators, BIBB, for polymerization. SI-ATRP is used to grafted the PNIPAM brushes on silica particles.

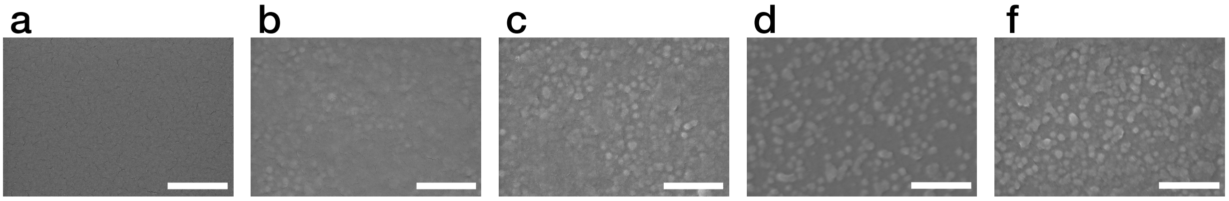


Supplementary Figure 2. **PNIPAM-grafted substrates.** SEM images of SS_PNIPAM (**a**), RS_0.22_PNIPAM (**b**), RS_0.36_PNIPAM (**c**), RS_0.46_PNIPAM (**d**), and RS_0.52_PNIPAM (**e**). Scale bars indicate 500 nm.

Supplementary Table I. **PNIPAM brush thickness of the PNIPAM-grafted substrates.** $h_{\mathrm{PNIPAM}}(20°C)$ is the swollen PNIPAM thickness and $h_{\mathrm{PNIPAM}}(40°C)$ is the collapsed PNIPAM thickness. Errors represent the standard deviations from five repeated measurements.

| | SS_PNIPAM | RS_0.22_PNIPAM | RS_0.36_PNIPAM | RS_0.46_PNIPAM | RS_0.52_PNIPAM |
|---|---|---|---|---|---|
| $h_{\mathrm{PNIPAM}}(20°C)$ (nm) | 35 ± 2 | 86 ± 5 | 83 ± 5 | 88 ± 4 | 85 ± 3 |
| $h_{\mathrm{PNIPAM}}(40°C)$ (nm) | 14 ± 1 | 35 ± 3 | 34 ± 2 | 35 ± 3 | 31 ± 2 |

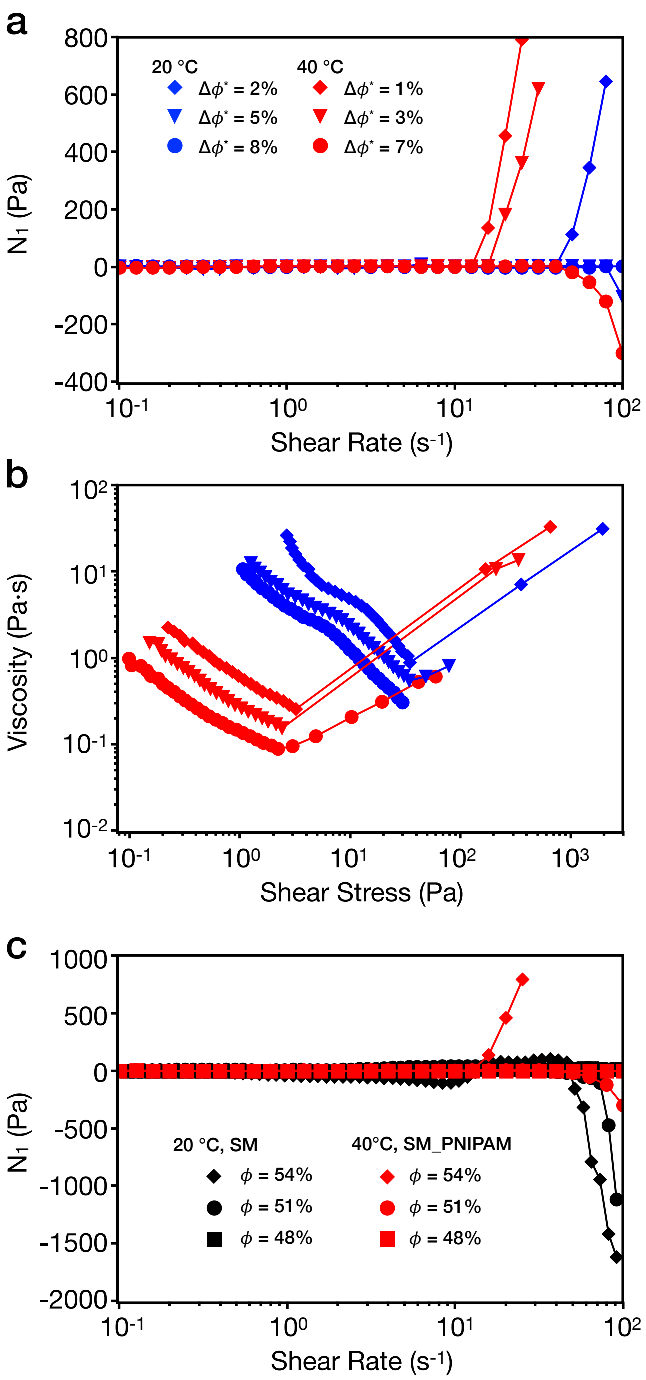


Supplementary Figure 3. **Shear-rheology experiments of PNIPAM-grafted smooth particles.** **a**, $N_1$ of the SM_PNIPAM flow curves shown in Figure 3b with the same legends. **b**, Flow curves from Figure 3b plotted as viscosity vs. stress with the same legends. **c**, $N_1$ of the normalized flow curves shown in Figure 3c with the same legends.

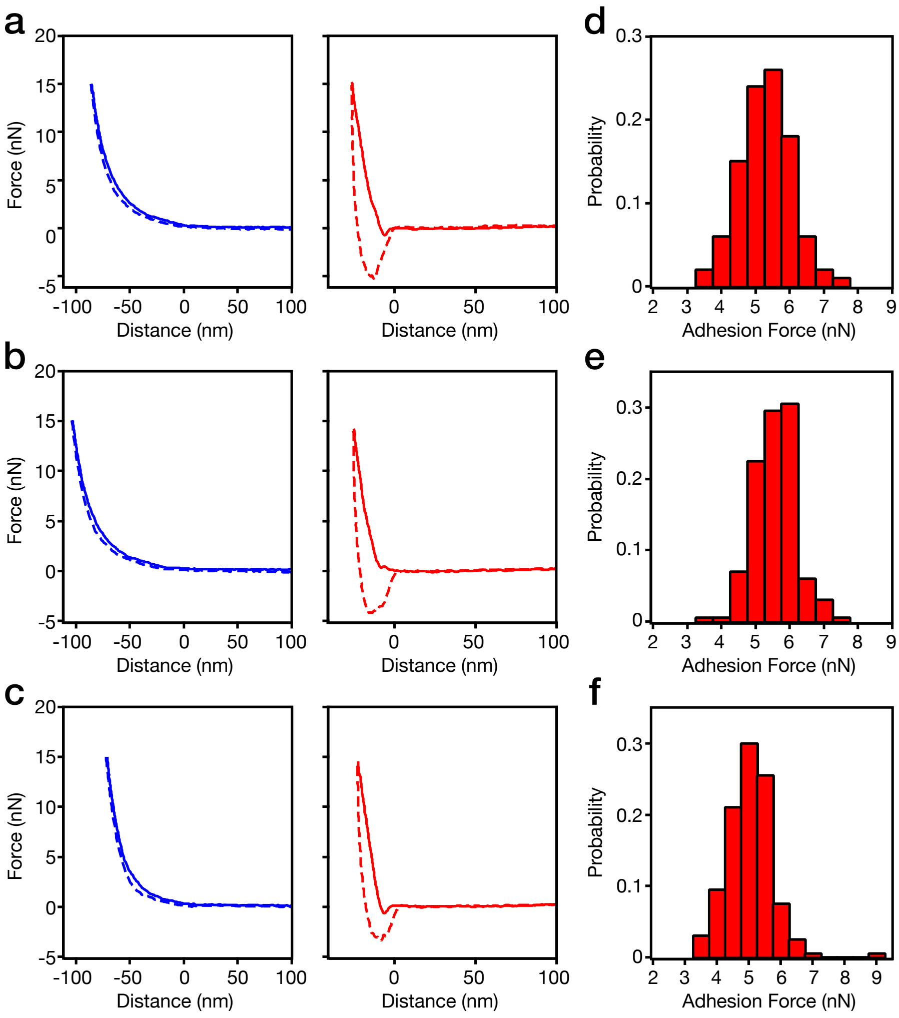


Supplementary Figure 4. **Force–distance curves of PNIPAM-grafted rough particles.** Approach (solid line) and retraction (dashed line) of the force–distance curves of the RB_0.22_PNIPAM system (**a**), RB_0.36_PNIPAM system (**b**), and RB_0.46_PNIPAM system (**c**) at 20 °C (left) and 40 °C (right). The adhesion forces of RB_0.22_PNIPAM system (5.6 ± 1.4 nN) (**d**), RB_0.36_PNIPAM system (5.5 ± 1.2 nN) (**e**), and RB_0.46_PNIPAM system (5.0 ± 1.3 nN) (**f**) measured at 40 °C. The bin width is 0.5 nN.

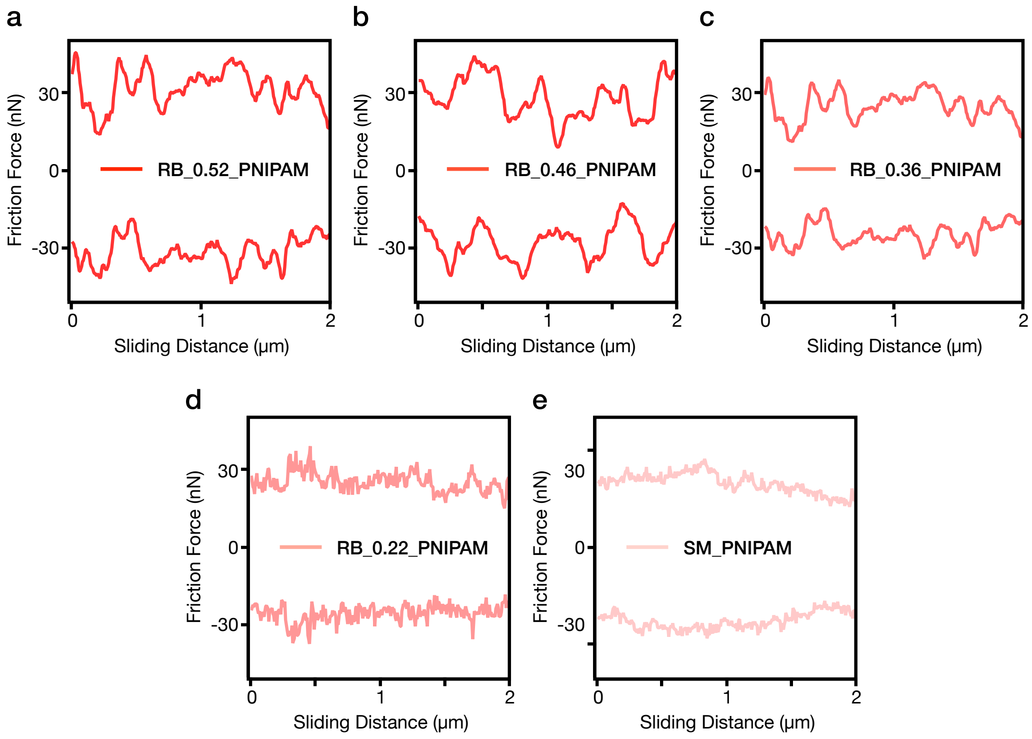


Supplementary Figure 5. **Friction loops of PNIPAM-grafted systems.** Friction loops of RB_0.52_PNIPAM (**a**), RB_0.46_PNIPAM (**b**), RB_0.36_PNIPAM (**c**), RB_0.22_PNIPAM (**d**), and SM_PNIPAM (**e**) at 60 nN and 40 °C.

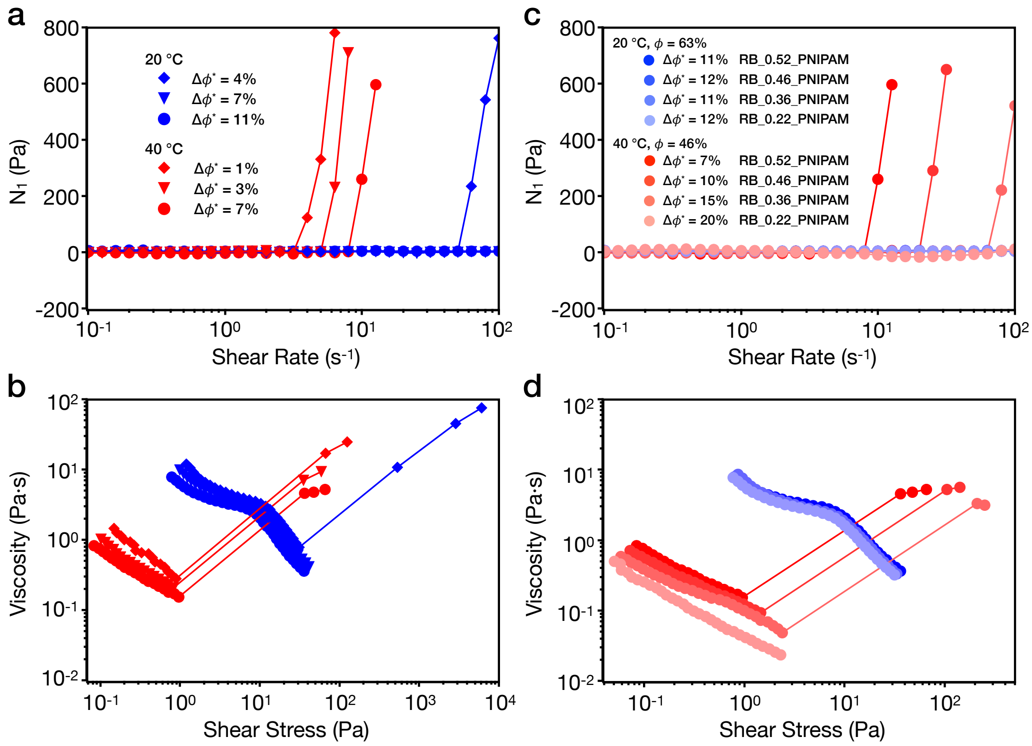


Supplementary Figure 6. **Shear-rheology experiments of PNIPAM-grafted rough particles.** **a**, $N_1$ of the RB_0.52_PNIPAM flow curves shown in Figure 5b with the same legends. **b**, Flow curves from Figure 5b plotted as viscosity vs. stress with the same legends. **c**, $N_1$ of the three PNIPAM-grafted rough systems shown in Figure 5c with the same legends. **d**, Flow curves from Figure 5c plotted as viscosity vs. stress with the same legends.

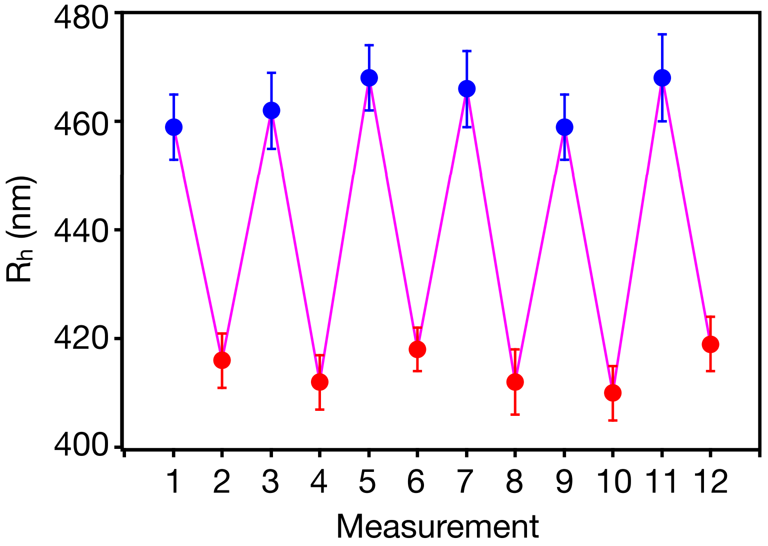


Supplementary Figure 7. **Thermo-switchable hydrodynamic radius of RB_0.52_PNIPAM.** The hydrodynamic radii ($R_h$) of the RB_0.52_PNIPAM particles at 20 °C (blue) and 40 °C (red). Error bars represent the standard deviations from ten repeated measurements.

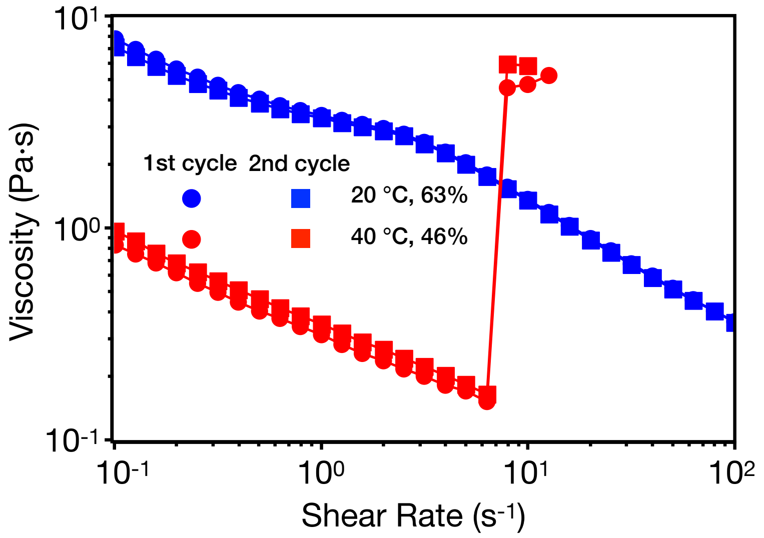


Supplementary Figure 8. **Reversible flow curves.** Two cycles of the flow curves of RB_0.52_PNIPAM systems with $\phi$ = 63% at 20 °C (blue) and $\phi$ = 46% at 40 °C (red).

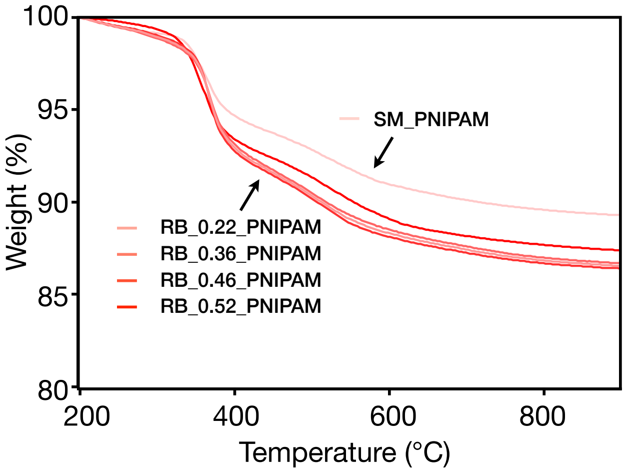


Supplementary Figure 9. **Determination of PNIPAM brush content.** TGA curves of SM_PNIPAM, RB_0.22_PNIPAM, RB_0.36_PNIPAM, RB_0.46_PNIPAM, and RB_0.52_PNIPAM.

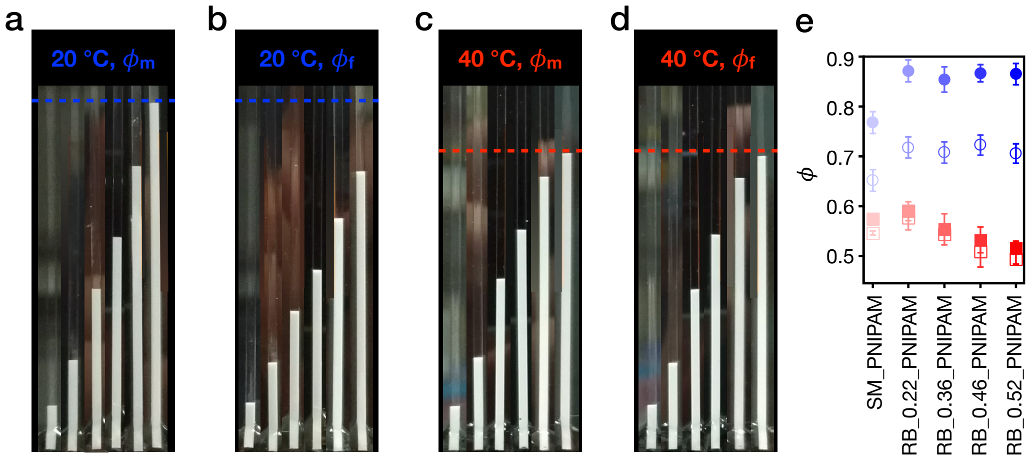


Supplementary Figure 10. **Centrifugation experiments and packing fractions.** Images of a particle suspension (RB_0.52_PNIPAM) (**a**), immediately after centrifugation at 20 °C for determination of $\phi_m$, (**b**), 10 days after centrifugation at 20 ° for determination of $\phi_f$, (**c**), immediately after centrifugation at 40 °C for determination of $\phi_m$, (**d**) 10 days after centrifugation at 40 ° for determination of $\phi_m$. The initial volume fraction $\phi_i$ increases from 9.7% (left) to 57.6% (right) for (**a**) and (**b**), and from 7.2% (left) to 42.3% (right) for (**c**) and (**d**). The blue dotted line in (**a**) and (**b**) marks the height of $\phi_i = 57.6\%$ immediately after centrifugation. The red dotted line in (**c**) and (**d**) marks the height of $\phi_i = 42.3\%$ immediately after centrifugation. (**e**), $\phi_m$ (open) and $\phi_f$ (filled) of the five colloidal suspensions at 20 °C (blue circles) and 40 °C (red squares). Error bars represent the standard deviations from four repeated measurements.
